# Structural and Mechanical Aberrations of the Nuclear Lamina in Disease

**DOI:** 10.3390/cells9081884

**Published:** 2020-08-11

**Authors:** Merel Stiekema, Marc A. M. J. van Zandvoort, Frans C. S. Ramaekers, Jos L. V. Broers

**Affiliations:** 1 Department of Genetics and Cell Biology, Maastricht University Medical Centre, NL-6200 MD Maastricht, The Netherlands; m.stiekema@maastrichtuniversity.nl (M.S.); mamj.vanzandvoort@maastrichtuniversity.nl (M.A.M.J.v.Z.); f.ramaekers@maastrichtuniversity.nl (F.C.S.R.); 2 GROW-School for Oncology and Developmental Biology, Maastricht University Medical Centre, NL-6200 MD Maastricht, The Netherlands; 3 CARIM-School for Cardiovascular Diseases, Maastricht University Medical Centre, NL-6200 MD Maastricht, The Netherlands; 4 Institute for Molecular Cardiovascular Research IMCAR, RWTH Aachen University, 52074 Aachen, Germany

**Keywords:** nuclear lamina, mechanosensing, mechanoresponse, nuclear rupture, nuclear stiffness, laminopathy, lamins in cancer, lamins and viruses

## Abstract

The nuclear lamins are the major components of the nuclear lamina in the nuclear envelope. Lamins are involved in numerous functions, including a role in providing structural support to the cell and the mechanosensing of the cell. Mutations in the genes encoding for lamins lead to the rare diseases termed laminopathies. However, not only laminopathies show alterations in the nuclear lamina. Deregulation of lamin expression is reported in multiple cancers and several viral infections lead to a disrupted nuclear lamina. The structural and mechanical effects of alterations in the nuclear lamina can partly explain the phenotypes seen in disease, such as muscular weakness in certain laminopathies and transmigration of cancer cells. However, a lot of answers to questions about the relation between changes in the nuclear lamina and disease development remain elusive. Here, we review the current understandings of the contribution of the nuclear lamina in the structural support and mechanosensing of healthy and diseased cells.

## 1. Introduction

The nuclear envelope (NE) is composed of a nuclear membrane, nuclear pore complexes (NPCs), and nuclear lamina. The nuclear lamina is a fibrous proteinaceous meshwork underlying the inner nuclear membrane. It is composed of the nuclear lamins and lamin-associated proteins [1,2,3,4]. The nuclear lamins are the main components of the nuclear lamina and together with the nuclear lamin-associated proteins, lamins are involved in a broad range of functions, for example, chromatin organization, DNA repair, and apoptosis [1,2,3,4,5]. The major mammalian lamins are lamin A, C, B1, and B2. Lamin A and C are classified as A-type lamins, and lamin B1 and B2 as B-type lamins. Three genes code for lamins, termed *LMNA*, *LMNB1*, and *LMNB2* [6,7].

*LMNB1* encodes lamin B1 and *LMNB2* encodes lamin B2, but also encodes lamin B3, a germ cell-specific isoform that arises by alternative RNA splicing. *LMNA* encodes lamin A, lamin C, and the germ cell-specific isoform lamin C2 and isoform lamin A∆10 [8,9].

Not long after the discovery of lamins, EM studies revealed in Xenopus a fine meshwork of filaments [10]. For decades it was assumed that such a meshwork was also the most prominent structure in human lamina formation. However, the characteristic meshwork appeared to be characteristic of Xenopus and could not be reproduced in vitro using purified human lamins. Additionally, the human lamins also form characteristic para-crystal structures [3]. As yet, the detailed composition of these crystals is not clear. Recent investigations using cryo-EM tomography showed that A- and B-type lamin form 3.5 nm thick filaments, consisting of tetramers [11,12].

The higher order lamin formation starts with the assembly of homodimers for every lamin subtype. Studies have shown that lamin A and lamin C form well-segregated homodimers [13]. The same holds true for lamin B1 and lamin B2 [14]. While tetramers can be made up of different types of lamin molecules, super-resolution microscopy, including PALM and dSTORM, has revealed distinct distribution patterns for A- and B-type lamins [15,16,17]. Similar distributions of lamin A and lamin B1 were found in the cryo-EM tomography study, confirming that sample preparations did not lead to major changes in the A- and B-type lamin organization in the nuclear lamina. The mammalian nuclear lamina could be studied with cryo-EM tomography by the use of ghost nuclei (i.e., vitamin-null mouse embryonic fibroblasts devoid of chromatin). This circumvents the obstacle of a too crowded environment around the nuclear lamina, which limits the ability to visualize the entire length of the lamina. The 3.5 nm thick A- and B-type lamin filaments are assembled into a ~14 nm thick meshwork, localized adjacent to the inner nuclear membrane, but underneath NPCs. In the lamin protofilament, the Ig-fold domains of the filaments appeared pairwise and in 10–20 nm steps along the rod of the filament [11,12].

**Figure 1:**
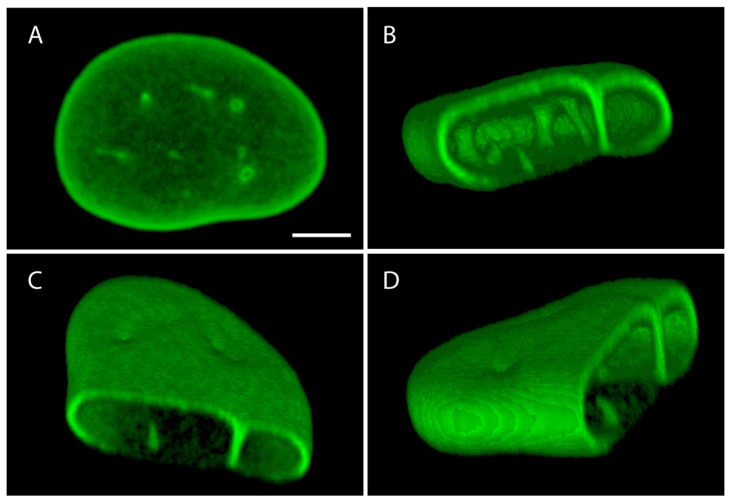
(**A–D**). Different views of 3-dimensional reconstruction using ImageJ 3-D viewer of the nucleus of a normal human dermal fibroblast cell, stained for lamin A/C using antibody Jol2 (kindly provided by C.J. Hutchison) in immunofluorescence. (**B**–**D**): In the 3-D image part of the signal was digitally removed in order to have a better insight into the nuclear interior. Note the prominent intranuclear channels, also called the nucleoplasmic reticulum, stained by the lamin A/C antibody. Scale bare represents 5 µm.

In the nucleoplasm, lamins assemble into structures like distinct foci, intranuclear and trans-nuclear channels, and nucleoplasmic veil-like structures (3D reconstruction in Figure 1) [18]. The intranuclear and trans-nuclear channels are thought to be part of the nucleoplasmic reticulum [19]. Nucleoplasmic A-type lamins have a higher mobility and are more soluble than peripheral A-type lamins, indicating a less polymerized form of A-type lamins in the nucleoplasm [9]. While the total thickness of the nuclear lamina in mouse embryonic fibroblasts does not exceed 15 nm [11,12], the large complexity ensures a plethora of functions assigned to this structure.

Mainly circumstantial evidence has led to a long list of potential functions of the lamins and their main structure, the nuclear lamina. First of all, evidence has accumulated that lamins have a key role in structural support, not only in maintaining nuclear strength and shape, but also in maintaining cellular integrity [20,21,22,23,24,25]. Next, studies have shown the importance of lamins in the reformation of the nuclear envelope after mitosis [9,26]. Furthermore, an intact lamina results in delay of the execution of apoptosis, showing the importance of A-type and B-type lamins in apoptosis [27]. Lamin deletion studies also revealed the impact of lamins on chromatin organization: loss of especially A-type lamins leads to disturbed heterochromatin organization [24,28,29]. This also holds true for *LMNA* mutant cells [30,31,32]. Lamins can interact directly with chromatin, or indirectly via other lamin-associated proteins. Heterochromatin is present at the nuclear periphery, where it can reposition certain genes to the nuclear lamina, thereby inducing transcriptional repression. Furthermore, A-type lamin deletion or downregulation can result in a decrease of heterochromatin and changes in chromosome territories. As a result, gene silencing mediated by heterochromatin formation becomes disturbed in these cells [33,34]. Lamins can also regulate gene expression by associating with several transcriptional regulators. Mutations and loss of lamins affects the intranuclear localization and stability of these transcriptional regulators and the affinity of lamins to certain binding partners [35]. Next to affecting chromatin organization, lamin mutations can also result in genomic instability by compromising DNA repair through long-range non-homologous end-joining (NHEJ) and homologous recombination (HR), and by affecting telomere structure and function [34].

While the importance of lamins in control of gene expression is becoming more and more evident (for a recent review, see, e.g., in [36]), this review will focus on another important aspect of lamins function, i.e., their role in structural support, mechanosensing, and mechanoresponse of cells. Their role in healthy cells is described below. Hereafter, we will discuss the alterations in the nuclear lamina in laminopathies, cancer, and viral infections. We will especially focus on the contribution of A-type lamins to the nuclear lamina, and the impact of absent or mutant A-type lamins on disease development.

## 2. Structural Support Provided by Nuclear Lamins

Lamins are classified within the IF superfamily, which led to the hypothesis that the lamins provide (mechanical) support to the nucleus [9]. Studies with Xenopus nuclear assembly systems confirmed this, as cell-free extracts depleted of lamins assemble in small and fragile nuclei [37]. This leads to nuclei that are prone to deform under physical compression (see below). Due to the role in providing structural support to the nucleus, lamins are highly dynamic during cell division. At the onset of mitosis, nuclear lamins, along with several other nuclear and NPC proteins, become phosphorylated by mitotic kinases, triggering disassembly of the lamina, the NPC, and the nuclear envelope [38]. The A-type lamins are solubilized and distributed throughout the cytoplasm. B-type lamins, however, stay insoluble and associated with the nuclear membrane, as they are farnesylated. In anaphase/telophase, the lamins are dephosphorylated and the NE reassembles around the segregated sister chromatids [9,26,39].

Several studies have underscored the importance of especially A-type lamins in maintaining physical strength, not only of the nucleus, but also load-bearing capacity of the complete cell. The tensegrity model by Ingber illustrated the potential importance of the different cytoskeletal elements in cellular and tissue strength [40]. Studies on the role of lamins showed that the nuclear lamina not only provides structural support, but also functions as a molecular shock absorber [41]. Later studies investigated the specific role of individual lamin proteins in maintaining the mechanical stiffness of nuclei. Lammerding et al. [42] compared the function of specific lamins (lamin A, C, and B1) in nuclear shape, stability, and stiffness in wild type mouse embryonic fibroblasts (MEFs) and mutated MEFs (lacking lamin A, both lamin A and C, or with a mutant form of lamin B1). It was found that lamins A and C are important contributors to the mechanical stiffness of nuclei, because in their absence, irregularly shaped nuclei and severely reduced nuclear stiffness were seen. Lamin B1 does not contribute to nuclear stiffness, but its absence leads to nuclear blebbing, suggesting a role in nuclear integrity. The suggestion that cells lacking *lmna* expression show an impact on nuclear stiffness was already shown by Broers et al. [20]. The mechanical stiffness of MEFs with normal *lmna* expression and no *lmna* expression was compared. This was tested by using a loading device for quantifying the mechanical resistance of a cell to compression and simultaneously observing the cells with confocal microscopy. This led to the result that cells lacking *lmna* expression have a significantly reduced mechanical stiffness. This was seen as a significantly lower resistance against indentation force. Furthermore, *lmna*^−^^/−^ cells needed a significantly lower force required for cellular bursting than *lmna*^+/+^ cells. Moreover, *lmna*^−/−^ nuclei showed isotropic deformation upon compression, while in *lmna*^+/+^ nuclei a strong anisotropic deformation was seen. This isotropic deformation was suggested to be caused by the lost connection with other cytoskeletal elements. Disruption of actin stress fibers and abnormal tubulin and vimentin organization were found in these cells [20]. A recent study by Wintner et al. [43] showed that not only lamin A, but also lamin B1, contributes to nuclear stiffness. Lamin A and lamin B1 were found to both equally render nuclear resistance when a force was continuously applied to the nucleus. However, nuclear viscosity was found to be mostly controlled by lamin A. These findings seem to contradict findings from a previous study of Lammerding et al. [42], in which lamin B1 was not found to contribute to mechanical stiffness of nuclei, but probably only to nuclear integrity. The difference in the conclusion of these studies might be explained by the different approaches; Lammerding et al. investigated the nuclear mechanics by analyzing the nuclear shape, nuclear dynamics over time, nuclear deformation under strain, and cell viability under prolonged mechanical stimulation, while Wintner used a viscoelastic model that captures dynamic resistance, lamin composition, phosphorylation states, and chromatin condensation.

A recent study investigated the role of vimentin in the mechanical properties of a cell and showed that not only lamins are important to protect the nucleus in mechanical stress, but also vimentin. It was found that vimentin is involved in mechanisms that regulate the shape of the nucleus, and that during migration through small channels, the absence of vimentin causes nuclei to undergo higher degrees of deformation, blebbing, and DNA damage in response to the compressive forces. Nuclear ruptures were found to occur more in vim^−/−^ cells than in vim^+/+^ cells, in both 2D and 3D substrates. In addition, in migration assays vim^−/−^ cells transit more readily through pores and contained more nuclei with blebs than vim^+/+^ cells. These nuclear blebs contained mainly a dilated lamin A meshwork and little if any lamin B. The expression levels of lamin A, B1/2, and C in vim^−/−^ cells, however, were found to be similar to those in vim^+/+^ cells. This indicated that the nuclear changes found in this study were a result from the role of vimentin and were independent of nuclear lamina effects [44]. It should be noted, however, that the extent and duration of the nuclear ruptures found in vim^−/−^ was much less severe than in *LMNA*^−^^/−^ cells.

The relation between loss of A-type lamins and altered actin cytoskeleton organization was studied by Corne et al. [45]. This study showed changes in protein levels that are involved in actin cytoskeleton organization as a result of induction of reduced A-type lamins in normal human dermal fibroblasts (nHDF). They found a direct link between the level of A-type lamins and focal adhesion (FA) formation, as proteins involved in FA were significantly downregulated in *LMNA* knockdown nHDFs. The downregulation of these proteins leads to decreased FA size, reduced initial cell attachment, increased cell migration, and increased cytoskeletal tension. Cells lacking A-type lamins have previously been found to affect cell migration, as a result of disturbed perinuclear cytoskeletal organization and reduced cytoskeletal stiffness [21,46].

Mechanical stress can lead to the loss of NE integrity, which results in NE rupture. An example of stress that can lead to NE rupture is confined cell migration. In NE rupture, the mechanical stress exerted onto the NE very likely triggers a local remodeling of the NE that is often accompanied by the formation of a nuclear extrusion or bleb; these blebs occur at sites of the NE with nuclear lamina defects. When a certain threshold level is reached the NE breaks, and this results in uncontrolled exchange of cytoplasmic and nuclear proteins, mis-localization of organelles, and DNA damage. Cells typically restore the NE shortly after the breakage. A new nuclear lamina is frequently formed in the bleb, this new lamina contains lamin A, but no lamin B1 [47,48,49,50]. The lamin A even accumulates at the rupture site, forming a “lamin scar” that remains visible for hours and presumably locally protects the nuclear membrane from further rupture [48].

## 3. Role of Lamins in Mechanosensing

Mechanosensing of cells occurs at the level of the extracellular matrix, which generates tensional force on the cell. Cells sense and respond to this via the cytoskeleton, which forms a structural chain between the cells’ environment and the nucleus [51]. The cytoskeleton ensures the maintenance of cell morphology and organization, while providing mechanical support for essential cellular functions [52]. Movement of organelles, such as the nucleus, requires forces generated by the cytoskeleton.

The nuclear lamina is physically connected to the linker of nucleoskeleton and cytoskeleton via a transmembrane complex (LINC complex), which transmits the forces generated by the cytoskeleton into the nucleoplasm [53]. This LINC complex is composed of a group of proteins in which Nesprin proteins appear to have a critical role. The Nesprin proteins penetrate the outer membrane of the NE and contain divergent *N*-termini and a *C*-terminal KASH (Klarsicht, ANC-1, Syne Homology) domain. The KASH domain interacts with the *C*-termini of SUN (Sad1, Unc-84) domain-containing proteins, which penetrate the inner membrane of the NE. The divergent *N*-termini of the SUN proteins are located in the nucleoplasm, interacting with the nuclear lamina and chromatin [47,51,53,54]. In this way, the inner and outer parts of the nucleus are connected and the force on the outer part can be translated into force on the inner part (and vice versa).

During nuclear movement, the KASH domain protein Nersprin-2G and SUN2 form so-called transmembrane actin-associated nuclear (TAN) lines, which are involved in the force transmission from the cytoskeleton into the nucleus. The TAN lines associate with dorsal actin cables that move towards the rear of the cell. A-type lamins anchor the TAN lines and thereby serve an essential role in the rearward nuclear movement and the force transmission that goes with it [53].

Several factors are involved in the association of TAN lines with the actin cables and are thus needed for nuclear movement. A component of the TAN lines, Samp1, associates with both SUN2 and lamin A/C. The maintenance of Samp1 presence at the nuclear envelope is dependent on the A-type lamins, indicating the importance of the A-type lamins in the nucleus–cytoskeleton connection. [55]. Furthermore, the diaphanous formin FHOD1 interacts with Nesprin-2G. FHOD1 strengthens the attachment between Nesprin-2G and the actin cable by providing a second site of interaction between Nesprin-2G and the actin cable [56]. Nuclear envelope-localized AAA+ (ATPase associated with various cellular activities) Torsin A and its activator, inner nuclear membrane protein lamin associated polypeptide 1 (LAP1), also contribute to the assembly of TAN lines and are found to be a key regulator of LINC complex-dependent nuclear–cytoskeletal coupling and actin retrograde flow [57].

The composition of the LINC complex and LINC complex-associated proteins varies per cell type. Different combinations of SUN- and KASH-domain proteins mediate different functions, including force transfer across the NE, and the stiffness of the cytoskeleton [47,54]. In order to sense and respond to environmental stimuli, all structural elements connecting the nucleus to the cell and its environment have to work together. If one of these structures is missing or mutated, this can change the way cells sense and respond to stimuli [58]. As A-type lamins are connected to components of the LINC complex, maintaining A-type lamins is of importance for maintaining proper mechanosensing.

A key role in the mechanoresponse could be exerted by the transcription factor Yes-associated protein (YAP), which is expressed in almost all cell types, including skeletal muscles. The YAP signaling pathway promotes target gene transcription and cell proliferation [59]. A-type lamins appear to regulate the localization and transcriptional activity of YAP, with reduced lamin levels being associated with increased YAP nuclear localization. The increased nuclear localization of YAP as seen in congenital muscle disorders due to *LMNA* mutations, is found to be a consequence of increased, deregulated nuclear import of YAP through the nuclear pore complexes [60].

In 3D cell migration, the nucleus is often the rate-limiting step, as it is the largest cellular organelle and it has a network of structural proteins. One of the mechanisms of migrating cells to navigate through tight spaces is to deform and squeeze through it. To do so, the morphology of both the cytoplasm and the nucleus has to be adapted. Lamins have been described to play a role in the structural and mechanical properties of the nucleus and thus also largely determine the deformability of the nucleus [61,62]. Cells in stiffer, mechanically stressed tissues have stiffer and stronger nuclei. As shown by Swift et al. [63], the lamin A level increases from soft to stiff tissue. Soft tissues, such as the brain and marrow, have a 30-fold lower lamin A level than stiffer tissues such as cartilage and bone. Additionally, cells grown on soft matrices show significantly more phosphorylation of lamin A. Lamin A phosphorylation is associated with increased solubility and degradation, which leads to minimized lamin A levels. This higher phosphorylation rate can be explained by better access of Ser/Thr kinase(s) to lamin A at low tension in the nucleus. The B-type lamins are constitutively expressed in different tissue stiffnesses. Another difference in A-type lamin expression in tensed cells is described by Ihalainen et al. [64]. This study showed that two N- and C-terminal lamin A/C epitopes exhibit apical-to-basal differences under environmental conditions known to upregulate cell contractility.

Pieuchot et al. termed the ability of cells to respond to cell-scale curvature variation “curvotaxis” and shows it is dependent on the actin cytoskeleton, the LINC complex, and the nuclear lamina [65]. Cell-scale curvature modulations do not only have impact on nuclear shape and cell migration, but also on gene expression.

Mutations in genes encoding for nuclear lamins or lamin-associated proteins can cause several diseases, for example, Emery–Dreifuss muscular dystrophy (EDMD), type II Dunnigan-type familial partial lipodystrophy, and the Hutchinson–Gilford progeria syndrome (HGPS) [5]. Lamins are also often dysregulated in cancer [66]. Furthermore, the nuclear lamina is disrupted in the viral entry and/or egress in several viral infections [67]. In the following sections we describe the structural and mechanical aberrations of the nuclear lamina in these mentioned types of diseases.

## 4. The Nuclear Lamina in Laminopathies

Mutations in genes encoding for lamins or lamin-associated protein can lead to several diseases, termed laminopathies. While these diseases are rare, the disease phenotypes are shared with many other more common ones, for example, insulin resistance and aging. Especially mutations in the *LMNA* gene cause these rare clinical disorders. Until now, more than 600 laminopathy mutations have been found in the *LMNA* gene, affecting many different tissues [5]. The laminopathies caused by mutations in the *LMNA* gene can be grouped into those that affect striated muscle, those that affect adipose tissue, those involving peripheral nerve systems, or those involving multiple systems with signs of accelerated aging. In this robust classification, there is some overlap between affected organs and tissues [8,68]. Next to mutations in the *LMNA* gene, encoding for A-type lamins, mutations in genes encoding for B-type lamins and lamin-associated proteins have been reported to cause diseases. Duplication or upstream deletion of lamin B1 can lead to leukodystrophy [69], while *LMNB2* mutations can lead to multiple different disease phenotypes [70,71].

How a mutation in lamins can lead to a variety in often tissue-specific disorders remains unclear. There are now three main hypotheses that are often described. The first one is the “structural hypothesis”, suggesting that *LMNA* mutations increase the fragility of the nucleus, leading to cell death and progressive failure in mechanically stressed tissues, such as muscle tissue. Another hypothesis is “the gene expression hypothesis”, which states that lamin mutations interfere with tissue-specific genes. The lamin mutation may inhibit binding to tissue-specific transcription factors or lead to abnormal gene activation or silencing during differentiation, which can lead to development of different disease phenotypes. The last hypothesis is about impaired stem cell function. It proposes that lamin mutations may cause abnormal differentiation or depletion of adult stem cells. One hypothesis does not exclude the other: laminopathies possibly arise from a combination of the different hypotheses [34,35].

### 4.1. Changes in Nuclear Morphology

**Figure 2:**
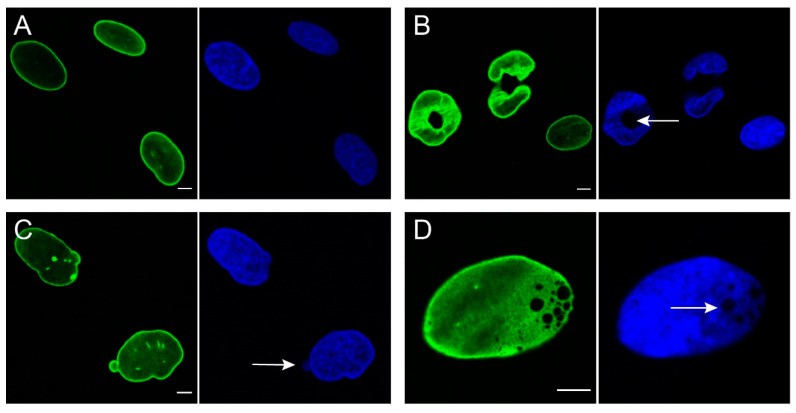
Nuclear abnormalities frequently observed in human dermal fibroblasts from laminopathy patients. Green: lamin A/C staining using Jol2, Blue: DAPI staining of nuclei. (**A**) normal nuclei; (**B**) donut-like shaped nuclei with irregular lamin A/C staining. Note the hole transversing the complete nucleus (arrow); (**C**) nuclear blebbing: nuclear herniations with increased lamin A/C expression. Sometimes, however, lamin A/C expression can be absent in blebs (not shown). Arrow indicates presence of low amount of DNA in bleb; (**D**) honeycomb structures, creating an intranuclear gap (arrow). Bars represent 5 µm.

The first cellular pathophysiological observation in laminopathies was an abnormal nuclear morphology due to mutations in the *LMNA* gene. Cells that lack or express abnormalities in A-type lamins can show different morphological changes, such as nuclear blebs (herniations, including micronuclei), honeycomb structures, donut-like structures, or combinations of the aforementioned categories (Figure 2), as categorized in a study of van Tienen et al. [72]. The morphological changes seen in *LMNA*-mutated cells differ upon cell type, lamin protein sequence, protein expression levels in transfected cells, and culture conditions [8]. Abnormal nuclei are not seen in all laminopathic cells. In the study of van Tienen et al. [72], fibroblast cultures were classified as laminopathic or normal by the detection of nuclear abnormalities. It was found that laminopathy classification based on a significant number of abnormal nuclei in fibroblasts is a quick and reliable method to confirm pathogenicity. The found percentage of abnormal nuclei in the laminopathic cells and control cells were in line with the study of Decaudain et al., which showed 15–25% dysmorphic nuclei in patients with a *LMNA* mutation and 5% abnormal nuclei in control fibroblasts [73]**.**

### 4.2. Changes in Structural and Mechanical Properties

A *LMNA* mutation does not only affect the cell morphology, but also has severe effects on the structural and mechanical properties of a cell [58]. Mechanical weakness of cells explains part of the symptoms seen in patients with laminopathies, as also described by the structural hypothesis [18].

#### 4.2.1. Nuclear Envelope Rupture

NE rupture was first described for cells with altered lamin expression; however, all cells regularly exhibit transient nuclear envelope rupture [47]. NE rupture as a result of lower lamin A expression, increased actinomyosin contractility, cell cycle migration in 3D, and increased stiffness of the ECM has the drastic consequence of leakage of nuclear DNA repair factors into the cytoplasm, resulting in DNA damage, telomere shortening, and cell cycle arrest [74,75]. It remains unclear if these nuclear lamina defects are caused by excessive nuclear deformation during migration or are pre-existing abnormalities in the nuclear envelope. However, when sites of the NE are more fragile, for instance, due to lamin mutations, the cell more easily reaches the threshold at which the NE will break, leading to higher NE rupture rates [48,49]. De Vos et al. [76] indeed showed more frequent NE ruptures in laminopathy patient fibroblasts in comparison to non-laminopathy patient fibroblasts. This was demonstrated by scrape loading cells with Texas Red-labeled dextran that should normally not pass the nuclear pores. The cells were observed with confocal microscopy, and after 24 h the cells with a *LMNA* mutation showed significantly more nuclear staining than healthy control cells. It was confirmed by live cell imaging using enhanced yellow fluorescent protein-tagged nuclear localization signal (EYPF-NLS) that this happens due to a larger structural defect in the nuclear lamina This showed an intranuclear EYFP-NLS signal in control cells, but sudden decrease in the EYFP-NLS intranuclear signal with an increase of the EYFP-NLS signal in the cytoplasm in laminopathy cells. It was confirmed by live cell imaging using enhanced yellow fluorescent protein-tagged nuclear localization signal (EYFP-NLS) that this happens due to a larger structural defect in the nuclear lamina. However, the ruptures were also detected in diseased nuclei without apparent morphological aberrations.

Furthermore, different *LMNA* mutations demonstrate variable NE rupture frequencies. Laminopathies with the most severe phenotype showed the most frequent nuclear ruptures. The ruptures were also observed in laminopathy cells with accumulation of partly processed or unprocessed mutant prelamin A. Moreover, cells with accumulation of the lamin A mutant progerin, as seen in HGPS cells, also displayed increased nuclear ruptures [76]. In these nuclear rupture events, nucleocytoplasmic compartmentalization is affected. Cytoplasmic proteins RelA and Cyclin B1 were found to enter the nucleus during rupture and intranuclear component octamer binding transcription factor 1 (OCT1) and nuclear structures containing the promyelocytic leukemia (PML) tumor suppressor protein could enter the cytoplasm. This nucleocytoplasmic compartmentalization affects the cellular function by altering gene expression, as found for OCT1 [76].

#### 4.2.2. Actin Cytoskeletal Organization Defects

Next to mechanical defects, mechanosensing and appropriate mechanoresponse appear to be affected in laminopathy cells. Several studies provided a link between A-type lamins mutations and actin cytoskeletal organization defects.

Tamiello et al. [22] investigated the effect of culturing diseased HDF on substrates with different stiffness. On substrates with stiffness higher than 10 kPa, malformations and rupture of the nuclei were observed. However, on a soft substrate (3 kPa), the nuclei of laminopathy cells experienced less mechanical stiffness. Under these conditions, the abnormal nuclear morphology of these *LMNA* mutant cells, as seen in normal culturing conditions, could be normalized and nuclear ruptures could be prevented. Less nuclear abnormalities in a soft substrate is related to the reduced cytoskeleton forces that the nuclear membrane is exposed to, as seen by decreased actin expression in these cells. In the *LMNA* mutant cells grown on the substrates with a stiffness higher than 10 kPa, aberrations in actin cytoskeleton organization were observed in ~5% of the cells. These aberrations included detachment of actin stress fibers in the perinuclear region with formation of a speckled pattern of actin, suggesting actin depolymerization in these areas. The nuclear abnormalities in *LMNA* mutant cells were found to have a direct correlation to the level of actin–cytoskeleton tension.

The effect of A-type lamins on actin stress fibers was further studied by Loosdregt et al. [58]. Actin cytoskeleton organization is the main determinant of cell shape, structure, and cellular stiffness [22]. Actin stress fibers provide cells the ability to exert contractile stress onto their environment, in order to, for example, migrate [58]. In the study, the difference in contractile stress generation and actin stress fiber organization between *lmna*^−/−^ cells and *lmna*^+/+^ cells was quantified with the thin film method and fluorescence microscopy. This resulted in a fivefold lower contractile stress generation by *lmna*^−/−^ cells in comparison to *lmna*^+/+^ cells. The lower contractile stress generation was associated with significantly less organized actin stress fiber organization with thinner actin fibers and smaller focal adhesions.

As described (Section 2), the disturbed cytoskeletal organization affects cell migration. The role of progerin expression in 3D cell migration was also studied. Progerin is a lamin A mutant seen in HGPS and is known to stiffen the nucleus. The cell migration in a 3D micropillar array of fibroblasts from a HGFP patient and a healthy adult control were compared. The interspacing of micropillars could be tuned at 6, 8, and 12 µm. The healthy cells were able to easily enter and exit the micropillar array, and thereby quickly adapting and changing shape. The HGPS cells, however, showed increased nuclear defects with decrease in interpillar space. Besides, less cells were able to enter the pillars with a decrease in the space [77]. This again confirms that nuclear deformability is an important factor in 3D cell migration.

#### 4.2.3. Signaling Pathway Alterations

A-type lamin mutations do not only affect the organization of lamins in the nuclear envelope, but also in the intranuclear structures and veil-like network in the nucleoplasm. The lamin mobility increases, which indicates loss of structural stability of lamin polymers [18].

Structural changes in A-type lamins can directly or indirectly affect multiple signaling pathways by altering the interactions of A-type lamins with signaling molecules. The nuclear translocation and downstream signaling of transcription factor MKL1 (important in cardiac development and function) was found to be impaired in lamin-A/C-deficient mutant cells. Nuclear import and export of MKL1 is regulated via changes in actin polymerization. A-type-deficient cells could therefore cause altered nucleocytoplasmic shuttling of MKL1 via the altered actin dynamics [78]. MKL1 regulates, through serum response factor, the expression of signaling molecules, transcription factors, and several cytoskeletal components [59].

**Figure 3:**
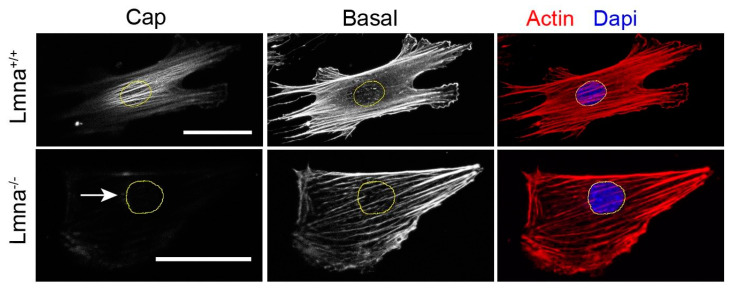
Impaired directional actin cap formation in *lmna*^−/−^ cells. Cells were seeded on oval microposts causing directed orientation. After staining for F-actin, confocal z-series were recorded, which show oriented actin fibers at bottom of the cell (basal) in both types of cells, along with an oriented actin cap in *lmna*^+/+^ cell. This cap on top of the cell is absent in the *lmna*^−/−^ cell (arrow). Bars represent 10 µm. Adapted from Tamiello et al. [79].

YAP is another signaling molecule that has been found to show aberrant regulation in muscle stem cells (MuSCs) of *LMNA*-related congenital muscular dystrophy (L-CMD) patients. As high YAP expression and activity promotes proliferation but prevents differentiation of myogenic cell precursors, it might be that an altered YAP expression in MuSCs also contributes negatively to skeletal muscle differentiation and thereby the physiopathology of lamin-related muscle dystrophy [60]. YAP furthermore exerts an important role in the mechanoresponse, as already described. Indeed, impaired mechanoresponse could be visualized at the level of (lack of) actin filament reorganization upon mechanical cues. In response to realignment of actin fibers, normally a nuclear actin cap should be formed allowing nuclei to reorient into the direction of the mechanical stress but *LMNA* knockout cells failed to do so (Figure 3) [79]. This also holds true for cells exposed to mechanical stretching. *LMNA*^+/+^ cells did form a more pronounced actin cap upon stretching, while *LMNA*^−/−^ did not do so [80].

In addition, Schwartz et al. [81] proposed that lamin mutations in human muscle cell precursors could directly or indirectly alter interactions of FHOD1 with Nesprin-2, with increased FHOD1 activity in soft matrix as a result. Increased FHOD1 activity could promote FHOD1-dependent F-actin formation, which in turn contributes to abnormal YAP activity and thus a defective mechanosensing response.

Another example of affected signaling pathways can be found in cardiomyopathy, where A-type lamin mutations (but also LINC protein SUN2 ablation) seem to be involved in an enhanced mechanoresponsive MAPK/AKT signaling, correlating with hypertrophy [82,83].

These examples of mis-regulation in signaling pathways are able to explain some disease symptoms and fit in the “gene expression hypothesis”, which is one of the three hypothesis that explain the tissue-specific consequences of laminopathies.

#### 4.2.4. Structural Defects Explaining Laminopathy Symptoms

The laminopathies EDMD and L-CMD both affect striated muscles; however, L-CMD symptoms appear much earlier and in higher severity. Recently, Bertrand et al. [84] showed a difference between these diseases on a structural level of the nuclear lamina, leading to either an impact on post-natal muscle development or muscle maintenance. L-CMD mutations affect post-natal muscle development through major defects in lamin A/C polymerization, with effects on the chromatin reorganization that is necessary for cell cycle exit and activation of myogenesis. EDMD mutations influence the muscle maintenance by weakening the nuclear lamina thereby making the cell more prone to DNA damage.

Another mutation on the structural level of the nuclear lamina is, for example, the 50-aa deletion in the *C*-terminus of the mutant lamin A (progerin) in HGPS. This deletion results in the stable farnesylation and carboxymethylation of progerin. As a consequence, progerin associates with the membrane during mitosis, delaying the onset and progression of cytokinesis and the targeting of NE components to daughter cell nuclei in late telophase/early G1. These mitotic defects can be linked to accelerated aging, a major symptom of HGPS [85].

As indicated, structural abnormalities in the nuclear lamina do not only lead to impaired physical strength of the nucleus in laminopathy cells, but lead also to loss of connections, needed for mechanosensing, transduction, and response. Lamin mutations do not only affect the lamina structure, but also have negative effects on the organization of proteins associated with the nuclear envelope. For instance, emerin is maintained at the nuclear envelope only in the presence of A-type lamins. Loss of A-type lamins, but also A-type lamin mutations, lead to displacement of emerin to the endoplasmic reticulum [86]. In addition, many nuclear envelope transmembrane proteins (NETs) depend on a correct organization of lamins and emerin to make up a proper functioning lamina. As more than 150 different NET proteins have been identified, it is very difficult to assess the impact of lamin mutations on the functioning of these proteins, especially as the function of most of them is unknown [87]. Moreover, both expression of LINC complex proteins, as seen for SUN and nesprin, as well as their localization can be disturbed due to lamin mutations, leading to defective nucleocytoplasmic connections (for a recent review, see [88]).

Moreover, improper chromatin organization due to *LMNA* mutations will lead to disturbed gene expression, bringing together the main hypotheses, why and how laminopathies develop (see also in [89]). As structure and function are so tightly connected, it will be very difficult, if not impossible to untwine these different functions of the nuclear lamina.

## 5. The Nuclear Lamina in Cancer

Several studies have described nuclear changes in neoplasia. These abnormal-shaped nuclei resemble those seen in laminopathy cells. This suggests a link between dysmorphic nuclei seen in cancer cells and altered expression of lamin proteins. Normally, proliferative cells are devoid of A-type lamin expression; many neoplastic tissues, however, do show A-type lamin expression. Conversely, cells that do not proliferate can be devoid of lamin expression [90]. The deregulation of lamin expression in a variety of cancers has been reported. A-type lamins are found to be overexpressed in ovarian cancers, colorectal cancer, and cancers of the skin and prostate [91,92,93,94,95]. However, next to overexpression of A-type lamins in colorectal cancer, also decreased levels have been reported in these cancers [96]. Lamin A levels are also reduced in rapid growth in basal cell carcinoma in skin cancers and A-type lamins are silenced in leukemias and lymphomas, gastric cancer, and small lung cancer [97,98,99,100]. Furthermore, A-type lamin expression is downregulated in breast cancer and gastric carcinoma [101,102]. While a trend is seen towards reduced A-type lamin expression along with loss of differentiation and increase in proliferation, not all studies on lamins in cancer show this correlation. It should be noted, however, that most studies were performed using a limited number of A-type lamin antibodies, not differentiating between lamin A and lamin C expression, while loss of lamin A can be accompanied by increase of lamin C in the same cells [93]. Many studies were performed on paraffin sections only, hampered by the reliability of staining reactions upon antigen retrieval. Clearly, much more research at different analysis levels (mRNA expression, single cell analysis, immunoblotting, etc.) should be performed to draw any firm conclusions. Despite these limitations, A-type lamin expression can be used as a marker to distinguish between tumor subtypes within a certain group of tumors with otherwise similar characteristics [90]. For example, small and non-small lung cell carcinoma cells can be distinguished by differences in A/C lamin levels [103].

Next to changes in lamin A/C expression levels, also their localization can be abnormal in tumors. For instance, nuclear aggregates of lamin A/C and aberrant cytoplasmic staining has been observed in gastrointestinal and lung carcinomas [99,104].

B-type lamins are more ubiquitously expressed than A-type lamins, but also show some deregulations [90]. For example, upregulation of B-type lamins has been identified in hepatocellular carcinoma and lamin B1 was reduced in colon carcinomas and adenomas and gastric cancers [99,105]. Next to changed lamin expression, downregulation and mutation of Nesprins are also found in many tumor cell types [62].

Lamins are thought to possibly affect cancer progression through a variety of mechanisms, including altered proliferation, signaling, and migration [34,35]. Cancer cells transmigrate across the endothelial layer and the underlying basement membranes of the vessel walls in intravasation and extravasation during metastasis. In this transmigration process, the cancer cells have to pass through narrow constrictions [106]. As described, the nucleus often forms the rate-limiting step in 3D cell migration. Cells with low levels of A-type lamins could lead to more deformable nuclei, which makes cells able to pass more easily through constrictions smaller than the size of the nucleus and thereby possibly facilitates extravasation and invasion of malignant cells [34,35]. This was, for example, shown in the study of Fu et al. [106], where the cellular and nuclear morphology associated with the transmigration process was investigated in highly metastatic human breast cancer cells growing in a microfluidics device. The deformation of the nucleus took on average 4 h, while deformation of the cytoplasm only took 30 min. With smaller microchannels a lower percentage of cells transmigrated through the channel. The highly metastatic cells were compared with poorly metastatic cells, which led to a significantly higher percentage of highly metastatic cells that transmigrated the microchannels at each channel dimension in comparison to the poorly metastatic cells. This suggests a higher capability to deform cells in more metastatic cells.

Furthermore, Harada et al. [107] studied MSC, U251 glioblastoma, and A549 lung carcinoma cells in migration through narrow pores of 3 µm in diameter, which mimics those of tumor tissues. U251 cells have the lowest level of lamin A (and are derived from the softest tissue) and showed the fastest 3D migration. Furthermore, lamin A overexpression was found to be correlated with a limited migration; lamin A knock-down, however, favored easier migration.

**Figure 4:**
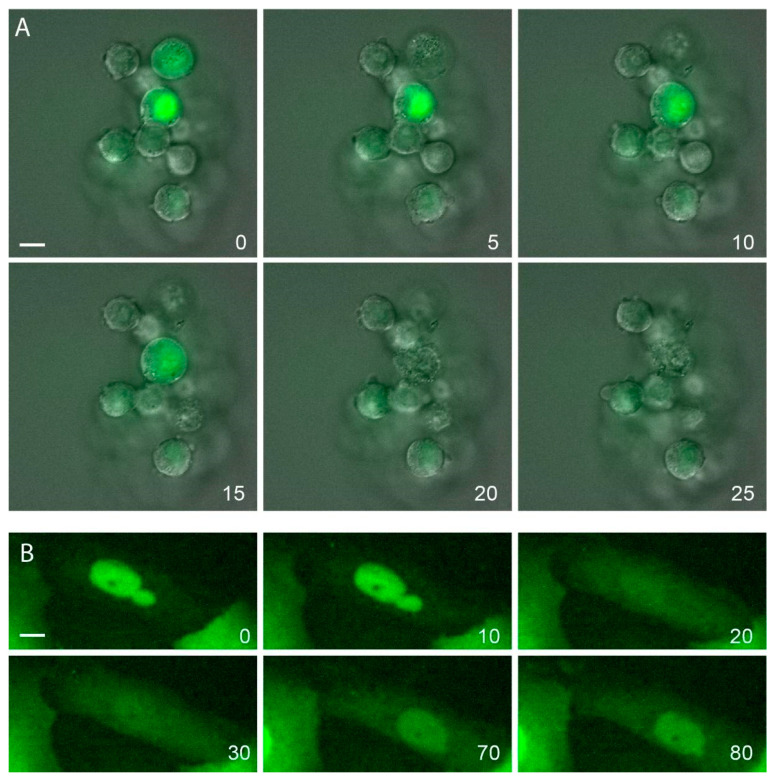
Time lapse recordings of nuclear ruptures in cancer cell lines, transfected with YFP-tagged NLS protein. (**A**) Small cell lung cancer cell line NL-SCLC3. Note that nuclear rupture at 10 min of recording leads to cell shrinkage and cell death (visible at 20 min, likely via apoptosis). (**B**) Skin cancer cell line MCC26, transfected with YFP-NLS. Note nuclear rupture around 20 min, with partial restoration of nuclear signal and without induction of cell death. Bars represent 10 µm.

As described, NE rupture rates are higher in situations with impaired lamin stability and nuclear deformation often occurs during confined cell migration. As human cancer cell lines often have altered lamin levels and transmigrate a lot during metastasis, NE rupture is more frequently seen in human cancer cells than in non-tumorigenic controls [48,50]. These ruptures can be irreversible, leading to cell death, as seen in Small Cell Lung Cancer cultures (Figure 4A), or reversible, with an apparently fully functional recovery of cells as seen in the human dermal cell lines MCC26 (Figure 4B).

A variety of cancer cells without known lamin defects or mutations also experience spontaneous NE rupture in the absence of external forces. This indicates the presence of other pathways that can induce NE rupture, for instance, forces within the nucleus (e.g., condensin II-mediated chromatin compaction) [49]. On the other hand, the loss of contractile actin bundles associated with the nucleus can inhibit NE rupture in cultured cancer cells [23].

The nuclear deformability and NE rupture during cell migration through confined spaces could result in genomic instability, particularly in proliferating cancer cells, which could further promote cancer progression [48,50]. The ESCRT-III subunits and the associated AAA-ATPase VPS4B are recruited to sites of NE rupture to help restore the NE integrity [48,50]. Selectively blocking one of these NE repair components could allow specific targeting cells prone to NE rupture, such as invasive cancer cells [48,50]. Another possible therapeutic approach to target cancer cells is blocking the NE rupture mechanism, which may reduce metastatic potential of migrating cancer cells [49].

As the lamin expression level varies among different cancer types and within tumors, it remains to be elucidated whether lamins modulate cancer metastasis by altering the mechanical properties of the nucleus or through effects of lamins on cell proliferation, signaling, and differentiation [35].

## 6. The Nuclear Lamina in Viral Infections

Many DNA viruses and some RNA viruses rely on the intranuclear genome replication and transcription machinery for productive viral replication [67]. The nuclear envelope acts like a barrier for nuclear egress. Viruses that cannot use the NPC have developed ways to overcome this barrier and exit the nucleus. To exit the nucleus without transversing the NPC, the NE has to be (partially) reorganized, which involves disassembly of the nuclear lamina. This partial reorganization leads to a nuclear morphology and nuclear behavior, reminiscent of laminopathies: heterochromatin gets disorganized and nuclear ruptures, similar to the ones observed in laminopathies can take place (see below).

### 6.1. Herpesviruses

The Herpesviridae family consists of several large DNA viruses. Viral genome replication, late gene transcription, and assembly of viral capsids take place in distinct foci named replication compartments (RCs). The structure and distribution of RCs in the nucleus are driven by interactions of viral DNA with viral and host proteins [108,109]. The NE acts as a barrier that prevents the large herpes viral nucleocapsids from leaving the nucleus. Due to the large size, the herpes viral capsids cannot be transported through the NPCs. Instead, the nucleocapsids bud through the inner nuclear membrane to perinuclear space. The primary envelope there fuses with the outer nuclear membrane and the nucleocapsids without envelope are released to the cytoplasm, where a new envelope is derived from the endoplasmic reticulum (ER) or Golgi apparatus membranes. To access the inner nuclear membrane disruption of the nuclear lamina is required, as the lamin filaments present a steric barrier to access the inner nuclear membrane [110,111,112,113]. The individual members of the Herpesviridae family have resemblances in the mechanisms of nuclear lamina disruption. There are some differences in the ways of interactions of the viral proteins with the nuclear lamina, but in the end it all results in nuclear lamina disruption [110]. Here, we only describe the lamina disruption in herpes simplex 1 (HSV-1) infection.

Disruption of the nuclear lamina by cells infected with HSV-1 involves the action of viral and host proteins. The viral transmembrane protein necessary for primary development, UL34, and the viral phosphoprotein UL31 form a complex that associates with the inner nuclear membrane. This complex has a role in primary envelopment of virions and is responsible for nuclear lamina disruption. Viruses lacking the UL34 or UL31 gene are not capable of inducing changes in the immunoreactivity of lamin A/C and lamin-associated polypeptide 2 (LAP2). Besides, the UL34 gene is required for disruption of lamin B. Both UL31 and UL34 can also bind lamin A/C directly, and overexpression of these proteins leads to partial lamin A/C relocalization [110,111]. Overexpression of UL31 leads to lamin A/C displacement from the nuclear rim into nucleoplasmic aggregates, and overexpression of UL34 leads to limited displacement of lamin A/C into the cytoplasm. When both proteins are co-expressed, lamin A/C is not dramatically displaced from the nuclear rim [111]. Next to nuclear lamina disruption, UL34 and UL31 have a role in characteristic nuclei enlargement [110].

Proteins homological to UL34 and UL31 were described for other herpesviruses: HSV-2, Pseudorabies virus, Human Cytomegalovirus, Murine Cytomegalovirus, and Epstein–Barr virus. These proteins are also responsible for primary envelopment and release of virions from the nucleus [110].

Viral serine/threonine kinase US3 colocalizes with UL34 and UL31 on the NE and associates with perinuclear virions. US3 phosphorylates UL34 and UL31 for the even distribution of the UL34-UL31 complex in the inner nuclear membrane. Furthermore, the correct localization of UL34 depends on the presence of lamin A/C. If US3 is absent or catalytically inactive, UL34 and UL31 are unevenly distributed throughout the nuclear rim. Furthermore, UL34 and UL31 accumulate in discrete foci located at the nuclear rim. In these regions, high concentration of UL31/UL34 exaggerate adjacent lamina perforations [110,114]. Therefore, in absence of US3, greater damage is done to the nuclear lamina, which suggests a negative regulation between US3 and the UL34-UL31 complex [110,114].

US3 also has a direct role in the nuclear lamina disruption by direct phosphorylation of lamin A/C on several sites in vitro and in vivo, by increasing lamin A/C solubility, and inducing defects in the nuclear lamina [110,114].

Another serine/threonine kinase that plays a role in nuclear lamina disruption is UL13. UL13 can phosphorylate US3, which leads to changes in the localization of UL34 and UL31 in the inner nuclear membrane. It is not clear whether UL13 influences the UL34–UL31 localization directly or via phosphorylation of US3 [110].

In the course of an HSV-1 infection, protein kinase C α (PKCα) and PKCδ are concentrated close to the NE. This localization is a result of its recruitment by UL34 and UL31 [114,115]. The even distribution of PKC along the nuclear rim is also regulated by US3. The protein kinases augment lamin B and emerin phosphorylation. The PKC activity is essential for HSV-1 infection as inhibition of all PKC isoforms causes substantial reduction of viral replication, accumulation of virus particles in the nuclei, and overall decrease in the number of viral capsids in the infected cells. However, specific inhibition of conventional PKCs or PKCδ does not inhibit viral replication. This suggests functional redundancy of these isoforms or involvement of other PKC forms in the viral life cycle [110,113,114].

Prior studies thus showed the importance of UL31 and UL34 for HSV nuclear egress, both via direct association with the nuclear lamina or indirect via other proteins. Regarding the possible role of other viral proteins, a lot is still unclear. The study of Wang et al. (2014), for example, demonstrated the involvement of cellular protein p32, which is probably targeted by the viral protein ICP34.5. They showed that ICP34.5 forms a complex with p32, in which p32 acts as a mediator of HSV-1 nuclear egress [116]. Furthermore, a study of Wu et al. (2016) suggests that the amino terminus of the γ_1_34.5 protein of HSV-1 is also linked to the disassembly process. However, the precise role of this protein is unknown [117]. Next to structural changes in the nuclear lamina itself, a HSV-1 viral infection also causes changes to lamin-associated proteins, such as lamin B receptor, emerin, and LAP2 [110].

### 6.2. Human Polyomaviruses

Thirteen human polyomaviruses have been identified to date [118]. Polyomaviruses are nonenveloped viruses that can infect dividing and non-dividing cells. These viruses are transported through the endosomal pathway to the ER, after that there are two models that describe the further transport to the nucleus [67,110,119]. One model involves changes in the nuclear lamina. It describes genome transfer into the nucleus through holes in the inner nuclear membrane, induced by caspase-6 cleavage of lamin A/C for local dissolution of the nuclear lamina. The exact mechanism behind this is yet unknown [119].

Next to lamin A/C, lamin B receptor (LBR) might be involved in nuclear egress of viral particles. Heterochromatin protein 1 (HP1) bound to the LBR is thought to contribute to reassembly of the NE after cell division. In the nuclear egress of human polyomavirus JC, the JCV agnoprotein (Agno) has been shown to induce HP1α dissociation from the lamin B receptor (LBR) [120]. The dissociation of HP1 from LBR is induced by the competitive binding of Agno to HP1, which subsequently leads to an increase in the lateral mobility of LBR and thus destabilization of the NE to facilitate virus egress [67,120]. Regarding the possible interactions of the agnoprotein with other proteins, there is a lot that is still unknown [121].

### 6.3. Baculoviruses

The nuclear egress of Baculoviruses is similar to that of herpesviruses, but much less details are known. As the nucleocapsids are 40–70 nm in diameter and 250–400 nm in length, modifying the nuclear lamina seems a necessary step in the budding process. Recently, it was confirmed that baculovirus infection also causes disruption of the nuclear lamina, as seen with herpesviruses [122]. This was shown by increased GFP-lamin B phosphorylation upon infection. GFP-lamin B was redistributed in the ring zone within the nuclei and associated with virions during baculovirus infection. The pathways belonging to the baculovirus-induced lamina disruption remains yet unknown [122].

### 6.4. Parvoviruses

Parvoviruses are small, nonenveloped, single-stranded DNA viruses. Both the entry and exit of the virus from the nucleus involve changes in the lamin structure. The parvoviruses are even small enough to enter the nucleus through the NPC, regulated by NLS. However, this is not the only way that these viruses enter the nucleus [67]. The nuclear entry of parvoviruses is also related to a nuclear envelop breakdown pathway [123,124]. Within this pathway, lamins become phosphorylated by PKCα and hyperphosphorylated by CDK-2. The (hyper)phosphorylation of lamins leads to lamin depolymerization, allowing entry and exit of large cargos from the nucleus by diffusion [124]. Other studies showed that after microinjection of the parvovirus Minute Virus of Mice (MVM), NE disruptions became visible as gaps in immunostaining of lamin A/C [123,125].

Nuclear egress of parvovirus capsids has been suggested to occur by active export via the NPC pathway prior to cell lysis. It has been shown that a canine parvovirus infection is accompanied with NPCs accumulation at the apical side of the nucleus along with a decrease in their overall density [126]. As NPCs are anchored into the nuclear lamina, changes in the distribution of NPCs correlates with nuclear lamina reorganization. This leads to regions rich in lamin B1 in the apical side together with an overall decrease of lamin A/C levels. These findings suggest a change in the organizational and/or functional status of nuclear lamins, rather than a degradation as seen in several other viruses [126].

### 6.5. Retroviruses

In HIV-infected cells, dynamic nuclear envelope disruptions, similar to the ones observed in laminopathy cells, were found. The protein Vpr induces localized disruption in the nuclear lamin architecture, with herniations in the nuclear envelope as a result. This intermittently led to the mixing of nuclear and cytoplasmic components, due to the rupture of the herniations.

The Vpr-induced NE herniations are thought to contribute to the cell cycle arrest in G2, observed in HIV-infected cells. G2 arrest increases HIV transcription, facilitating viral replication [127].

It is tempting to speculate on the impact of viral infection on tissue damage. Cells infected by a virus will lose mechanical strength due to the degradation of the nuclear lamina. As a result, the NE is more prone to rupture when exposed to mechanical stress, for example, during cell migration. The drastic consequence can be the uncontrolled exchange of cytoplasmic and nuclear proteins, mis-localization of organelles, and DNA damage. Unrepaired DNA damage could in turn result in the further development of diseases. In this respect, we are studying the effect of viral infections on heart function in cardiomyopathy patients. We predict that viral infections in these patients will aggravate the loss of cardiac function, initiated by a *LMNA* mutation, to a large extent.

## 7. Conclusions and Recommendations

The multiple functions of lamins and the nuclear lamina make it very difficult to assess the impact of lamina abnormalities on diseases initiated by *LMNA* mutations. Often, the disease phenotype cannot be directly linked to (loss of) function of the lamin polymers. In laminopathies, the direct loss of nuclear strength, combined with a loss of mechanosensing and response, does seem to be responsible for a large part of the observed phenotypes. Abnormalities due to deregulated gene expression are difficult to assess. A main question is what came first? Does altered gene expression cause structural and functional abnormalities or vice versa? The impact of specific *LMNA* mutations on disease development is also still largely unknown. Why do family members with the same *LMNA* mutations develop different laminopathies? A better knowledge about the underlying mechanism could lead to improved treatment regimens of laminopathies, which until now are mainly aimed at preventing further damage.

The role of lamins in diseases, in which no lamin mutations are involved, remains to a large extent speculative: Are lamins only targets of deregulation, or does altered lamin expression lead to more aggressive disease development? Clearly, at the level of cancer development and the level of viral infections a lot of additional research is needed to draw any conclusions on cause versus effect. Even without this knowledge, however, modulation of *LMNA* expression could decrease metastatic potential of cancer cells, or decrease efficiency of virus production in infected cells.
